# Barriers and enablers to physical activity in type 1 diabetes: A mixed‐methods study of adults and parent‐reported child perspectives in the UK

**DOI:** 10.1111/dme.70378

**Published:** 2026-06-19

**Authors:** Emma J. Cockcroft, Chris Bright, Hilary Nathan, Dan Farrow, Catherine Russon, Richard M. Pulsford, Robert. C. Andrews

**Affiliations:** ^1^ Department of Health and Community Sciences University of Exeter Medical School Exeter UK; ^2^ BreakthroughT1D London UK; ^3^ Department of Public Health and Sports Sciences University of Exeter Medical School Exeter UK; ^4^ Department of Clinical and Biomedical Sciences University of Exeter Medical School Exeter UK; ^5^ Department of Diabetes Taunton and Somerset NHS Foundation Trust Taunton UK; ^6^ NIHR Exeter Biomedical Research Centre (BRC) Exeter UK

**Keywords:** diabetes, exercise, health behaviour, intervention development, stigma

## Abstract

**Aims:**

To identify and compare barriers and enablers influencing physical activity in adults and parent‐proxy responses for children with type 1 diabetes in the UK.

**Methods:**

A cross sectional survey was distributed via BreakthroughT1D (formerly JDRF) networks between December 2022 and January 2023. The survey included closed and open‐ended questions on physical activity behaviours and influencing factors. Responses were collected from adults with type 1 diabetes and parent‐proxy responses for children with type 1 diabetes. Quantitative data were analysed using descriptive statistics and two‐proportion *z*‐tests. Free‐text responses were analysed thematically. Barriers and enablers were synthesised using the COM‐B model and socioecological framework to identify behavioural determinants and intervention targets.

**Results:**

311 responses were analysed (182 adults; 129 parent proxies for children). Stigma and negative comments were reported by nearly three‐quarters of both groups. Adults more frequently reported clinical and motivational barriers: fear of hypoglycaemia (53%) and lack of motivation (39%). Adults reported significantly less healthcare provider support for physical activity (24%) compared with parent‐proxy responses for children (57%). Parent‐proxy responses for children more frequently reported external barriers, including lack of education among coaches (31%).

**Conclusion:**

This is one of the first UK‐based studies to comprehensively compare barriers and enablers across age groups. Stigma in physical activity and sports settings emerges as a major, under‐recognised barrier requiring urgent attention. Multilevel interventions are required, targeting individual knowledge and motivation alongside systemic changes. These should include mandatory diabetes education for coaches and teachers, policy enforcement to address discrimination and enhanced healthcare provider support for physical activity.

**Summay**

**What is already known?**

People with type 1 diabetes face unique barriers to physical activity, including fear of hypoglycaemia and the complexity of diabetes management; existing research typically focuses on either adults or children in isolation and gives limited attention to social and environmental influences.

**What has this study found?**

This UK‐based study provides a comparative perspective across adults and parent‐proxy reports for children, identifying stigma and inadequate support in sport and physical activity settings as key, under‐recognised barriers, with differences by age group.

**What are the implications of this study?**

The findings highlight the need for multilevel interventions and policy action to reduce stigma and improve diabetes‐related knowledge and support among coaches, teachers, and healthcare professionals.

## 1 INTRODUCTION

Type 1 diabetes is a chronic autoimmune condition that affects 9.5 million individuals globally,^1^ with significant prevalence among both children and adults.^2^ Despite well‐documented benefits of physical activity,^3^, ^4^ people with type 1 diabetes engage in lower levels than their peers without the condition. For children and adolescents with type 1 diabetes, less than half meet the recommended 60 min of moderate‐to‐vigorous physical activity daily.^5^ Adults with type 1 diabetes also report lower engagement in structured exercise and recreational activities,^6^ with only around one‐third meeting recommendations of ≥ 150 of moderate‐to‐vigorous physical activity per week.^7^, ^8^ This disparity underscores the existence of unique challenges tied to type 1 diabetes management during physical activity, such as fears of hypoglycaemia, the need for precise insulin adjustments and logistical concerns related to monitoring blood glucose levels.^9^, ^10^, ^11^ This is in addition to commonly cited barriers to physical activity at a whole population level such as environmental factors, time and social norms.^12^

Theoretical models provide a structured lens for understanding and changing health‐related behaviours. The capability, opportunity and motivation model of behaviour (COM‐B) conceptualises behaviour as the interaction between capability, opportunity and motivation,^13^ providing a framework to identify modifiable targets for intervention. The socioecological model^14^ complements this by recognising that behaviour is shaped across multiple levels, including interpersonal, organisational, community and policy environments. Integrating these frameworks enables consideration of both individual and wider structural influences on physical activity.

Despite growing awareness of challenges relating to physical activity in type 1 diabetes, limited understanding remains of how barriers and motivators interact across groups and settings. Previous research has examined these factors in adults and children but has largely been conducted outside the UK and often relies on predefined questionnaire tools.^11^, ^15^, ^16^ Differences in healthcare systems, access to diabetes education and physical activity environments mean that findings from non‐UK settings may not be directly transferable to the UK context. As such, there remains a need for UK‐based research that directly compares experiences across age groups while capturing a broader range of lived experiences.

Most existing studies have relied on predefined questionnaire tools such as the barriers to physical activity in type 1 diabetes (BAPAD‐1) scale,^15^, ^16^ which, although validated in adults, have not been formally validated for use in children and adolescents and may restrict responses to predetermined barriers, potentially overlooking important context‐specific or emergent factors. Furthermore, while stigma in diabetes management has been increasingly recognized as an important issue affecting self care and disease management,^17^, ^18^, ^19^ its specific manifestation and impact within physical activity and sports settings remain under‐explored, particularly at scale. Previous studies, including qualitative and mixed‐methods research in both adults and children with type 1 diabetes,^9^, ^11^ have highlighted experiences of stigma and stereotyping in relation to physical activity. These include negative perceptions of capability, exclusion from sport and misunderstanding from peers, educators and activity providers. Recent qualitative work has further explored these experiences across the lifespan, describing challenges such as institutional misunderstanding, social exclusion and the need for more inclusive and supportive physical activity environments.^20^ However, this work has primarily taken a qualitative approach and has not quantified the prevalence or distribution of these barriers, nor directly compared patterns between adults and younger populations.

This study addresses these gaps through a survey exploring the lived experiences of adults and parent‐proxy responses for children with type 1 diabetes in the UK. By combining quantitative and qualitative data, it quantifies the prevalence of barriers and enablers and enables comparison across age groups. The use of open‐ended questions allows for identification of emergent and context‐specific factors beyond predefined checklists. Findings are interpreted using the COM‐B and socioecological frameworks to provide a theoretically informed understanding of how barriers and enablers operate across individual, interpersonal, organisational, community and policy levels.

## 2 METHODS

This study presents a secondary analysis of anonymised survey data exploring physical activity experiences in people with type 1 diabetes. The survey was developed and hosted by nfp research (https://nfpresearch.com) in partnership with Breakthrough T1D UK (formerly juvenile diabetes research foundation [JDRF]), the UK arm of the global charity Breakthrough T1D, which is dedicated to curing type 1 diabetes and improving the lives of those affected by the condition. No new data were collected; analysis remained within the originally agreed scope. According to institutional and national guidelines, secondary analysis of anonymised data did not require further ethical approval or consent. The research adhered to the declaration of Helsinki and institutional policies.

Participants rated statements about physical activity using a five‐point Likert scale, with additional multiple‐choice and free‐text items capturing motivations, barriers, stigma experiences and information sources. Open‐ended free‐text questions were included because checklist‐based tools (e.g. BAPAD‐1) restrict responses to predefined barriers, which may miss context‐specific or emergent factors. This approach enabled participants to describe lived experiences and emergent themes in their own words. The full survey is provided in the Supporting Information.

### 2.1 Sample

Survey data were collected from Dec 2022 to Jan 2023. The survey was distributed by Breakthrough T1D (formerly JDRF) via their network, inclusive of social media. The participants were a convenience sample of willing participants. All people in the UK with type 1 diabetes were eligible to participate. Because of the nature of recruitment efforts, it is not possible to determine the total number of potential participants approached to complete this survey.

Parent‐proxy responses for children were used for pragmatic reasons: It was not feasible to directly survey a large, nationally‐distributed sample of children and adolescents with type 1 diabetes via an online questionnaire, both due to ethical constraints and low response rates observed in direct‐to‐child survey studies. Engaging parents as respondents enabled effective, ethical and broad recruitment across the UK and ensured inclusion of children of all ages, particularly younger children who could not self‐complete the survey. Parents were asked to complete the survey either on behalf of their child or together with their child, depending on the child's age and ability to respond. No restriction was placed on which parent completed the survey, and no demographic data were collected on the responding parent. This pragmatic strategy is widely used and accepted in research with paediatric populations,^21^ but it is acknowledged that parent responses may not fully reflect their child's own experiences.

### 2.2 Data analysis

Survey responses were summarised using descriptive statistics. Continuous variables are presented as means (±SD) and categorical variables as percentages. Analyses were conducted in python 3.9 (Jupyterlab v3, Anaconda 2023). Two‐proportion *z*‐tests were used to compare groups (adults vs. parent‐proxy responses for children, and technology users vs. non‐users, including closed‐loop use). Multiple comparisons were addressed using the Benjamini–Hochberg false discovery rate procedure.

Free‐text responses were analysed thematically in NVivo (v12), following Braun and Clarke's approach.^22^ One researcher initially coded the responses, grouping similar codes into broader categories, which were refined in discussion with the wider team.

Quantitative and qualitative findings were synthesised using the COM‐B model and mapped onto the socioecological framework. This multilevel, theory‐informed approach enhances the identification of intervention targets spanning individual, interpersonal, organisational, community and policy levels.

### 2.3 Patient and public involvement

This study was co‐designed and delivered in collaboration with Breakthrough T1D UK, a national diabetes charity. People with lived experience of type 1 diabetes were involved throughout the project, including shaping survey content, interpreting findings and identifying practical implications. The research team includes individuals with direct personal experience of type 1 diabetes, ensuring that analysis and recommendations were grounded in real‐world relevance and priorities.

## 3 RESULTS

### 3.1 Sample characteristics

A total of 311 people responded to the survey, including 182 adults and 129 parent‐proxy responses for children (< 18 years). Respondents were geographically distributed across all UK nations and regions, with most identifying as white British (82%). The sample was 47% male, 52% female and < 1% non‐binary. Use of diabetes technology was common, with 60% using CGM and 53% using insulin pumps. Age was approximately normally distributed (skew = 0.33), with a mean (SD) age of 30.3 (18.2) years. Full demographic characteristics, including disease duration, gender, technology use, ethnicity and location, are summarised in Table 1.

**TABLE 1:** Characteristics of survey respondents.

		Total sample	Adults	Parent‐proxy responses for children
Total (*n*, %)		311	182 (59%)	129 (41%)
Age (mean, SD)		30.3 ± 18.2	42.9 ± 12.5	12.4 ± 5.0
Age at diagnosis			22.6 ± 14.5	
Years since diagnosis		11.2 ± 9.4	16.0 ± 9.1	4.4 ± 4.3
Gender	Male	146 (47%)	71 (39%)	75 (58%)
	Female	161 (52%)	110 (60%)	51 (39%)
	Non‐binary	2 (< 1%)	1 (1%)	1 (1%)
	Not reported	2 (< 1%)	‐	2 (2%)
Use of technology	Pump	164 (53%)	90 (49%)	74 (57%)
	Closed loop	75 (24%)	47 (26%)	28 (22%)
	CGM	186 (60%)	89 (49%)	97 (75%)
	Any technology	230 (73%)	121 (66%)	109 (84%)
Ethnicity (% white British)		254 (82%)	152 (84%)	102 (79%)
Geographical location	East midlands	13 (4.1%)	7 (3.8%)	6 (4.7%)
	East of England	20 (6.3%)	10 (5.5%)	10 (7.8%)
	London	36 (11.4%)	25 (13.7%)	11 (8.5%)
	North east	15 (4.8%)	11 (6.0%)	4 (3.1%)
	North west	30 (9.5%)	19 (10.4%)	11 (8.5%)
	Northern Ireland	9 (2.9%)	4 (2.2%)	5 (3.9%)
	Scotland	33 (10.5%)	28 (15.4%)	5 (3.9%)
	South east	64 (20.3%)	34 (18.7%)	30 (23.3%)
	South west	28 (8.9%)	12 (6.6%)	16 (12.4%)
	Wales	11 (3.5%)	5 (2.7%)	6 (4.7%)
	West midlands	16 (5.1%)	7 (3.8%)	9 (7.0%)
	Yorkshire/Humberside	17 (5.4%)	6 (3.3%)	11 (8.5%)
	Other	15 (4.8%)	11 (6.0%)	4 (3.1%)

Participants reported taking part in a variety of activities, with football, walking and swimming being the most common in parent‐proxy responses for children and walking, running and gym attendance being the most cited in adults. Data suggest adults more often reported individual/self‐paced activities (15 of top 20 reported activities) than in parent‐proxy responses for children (6 of the top 20 reported activities), who were more likely to report team sports/activities.

### 3.2 Attitudes, knowledge and self‐efficacy for physical activity

Adults were more likely to want to engage in more physical activity, with 65% of adults strongly agreeing or agreeing to the statement ‘I would like to engage in more physical activity’ compared with 42% of parent‐proxy responses for children. Both adults and parent‐proxy responses for children (98% and 91%, respectively) either strongly agreed or agreed that it is important for people living with type 1 diabetes to engage in physical activity.

Motivations for physical activity varied significantly between adults and children as reported by their parents (see Figure 1). While health and wellbeing were the most commonly reported drivers overall, adults were significantly more likely than children as reported by their parents to be motivated by general health (78.0% vs. 58.9%, *p* < 0.001, adjusted *p* = 0.001), mental health benefits (69.2% vs. 51.2%, *p* = 0.001, adjusted *p* = 0.002), glucose management (46.2% vs. 25.6%, *p* < 0.001, adjusted *p* = 0.001), body image concerns (36.3% vs. 17.8%, *p* < 0.001, adjusted *p* = 0.001), daily‐life needs (18.1% vs. 6.2%, *p* = 0.002, adjusted *p* = 0.004), financial or career goals (8.5% vs. 1.6%, *p* = 0.004, adjusted *p* = 0.006) and feelings of guilt (‘I should be active’) (16.5% vs. 6.2%, *p* = 0.006, adjusted *p* = 0.009).

**FIGURE 1:**
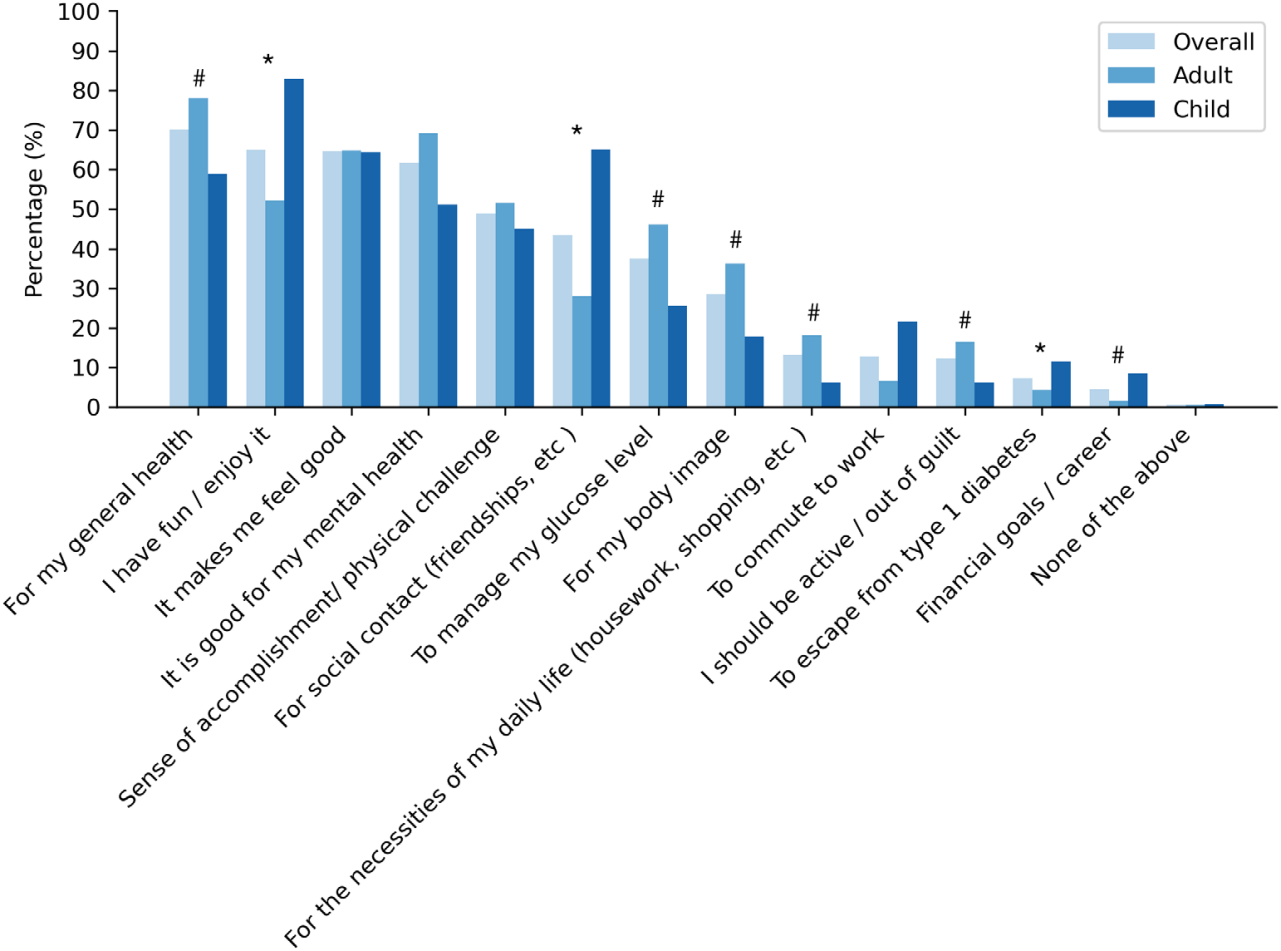
Participant reported reasons for engaging in physical activity. Significant differences between adults and parent‐proxy responses for children are shown with a # where adults were more likely to report motivation reason and * where parent‐proxy responses for children were more likely to report reason.

Children as reported by their parents were significantly more likely to be motivated by fun and enjoyment (82.9% vs. 52.2%, *p* < 0.001, adjusted *p* < 0.001), social contact (65.1% vs. 28.0%, *p* < 0.001, adjusted *p* < 0.001) and escaping from type 1 diabetes (11.6% vs. 4.4%, *p* = 0.016, adjusted *p* = 0.021) compared with adults.

In terms of knowledge and understanding, 30% of adults and 34% of children as reported by their parents felt they did not know enough about physical activity, and 42% and 38% of adults and children, respectively, would like to be more informed about how they could engage in physical activity. Additionally, 63% of adults and 65% of children felt that the knowledge of type 1 diabetes in those organising exercise is not sufficient to support them.

### 3.3 Barriers to engaging in physical activity

Barriers to physical activity reported by participants are shown in Figure 2 (individual‐level factors) and Figure 3 (external factors). The most frequently endorsed barrier was fear of hypo‐/hyper‐glycaemia (46.0%), followed by complexity of diabetes management (39.2%), insufficient time to exercise (33.4%) and lack of motivation (30.9%). Less common barriers included cost (12.2%), never having built an exercise habit (10.6%) and not enjoying exercise (8.7%). When comparing adults and parent‐proxy responses for children, three barriers differed significantly. Adults were more likely to report fear of hypo‐/hyper‐glycaemia (52.7% vs. 36.4%, *p* = 0.004, *adjusted p*‐value = 0.018) and lack of motivation (38.5% vs. 20.2%, *p* < 0.001, *adjusted p*‐value = 0.006).

**FIGURE 2:**
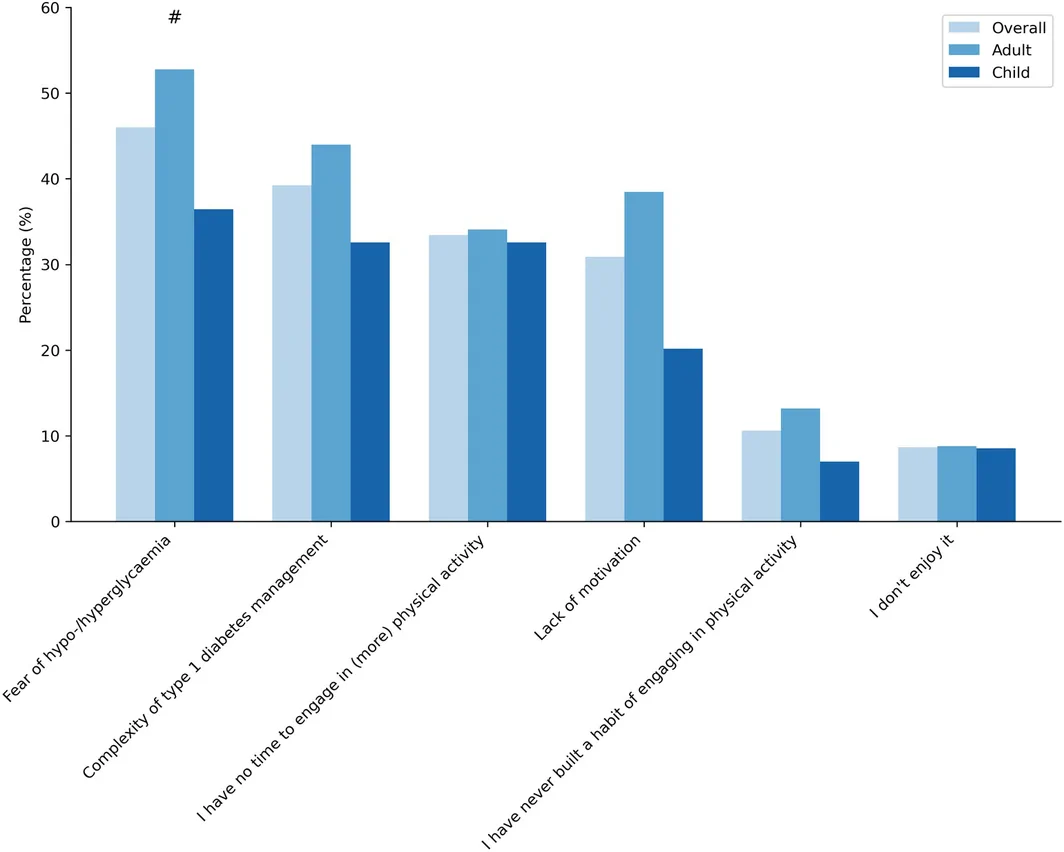
Indiviudal level barriers to physical activity. Significant differences between adults and parent‐proxy responses for children are shown with a # where adults were more likely to report motivation reason.

**FIGURE 3:**
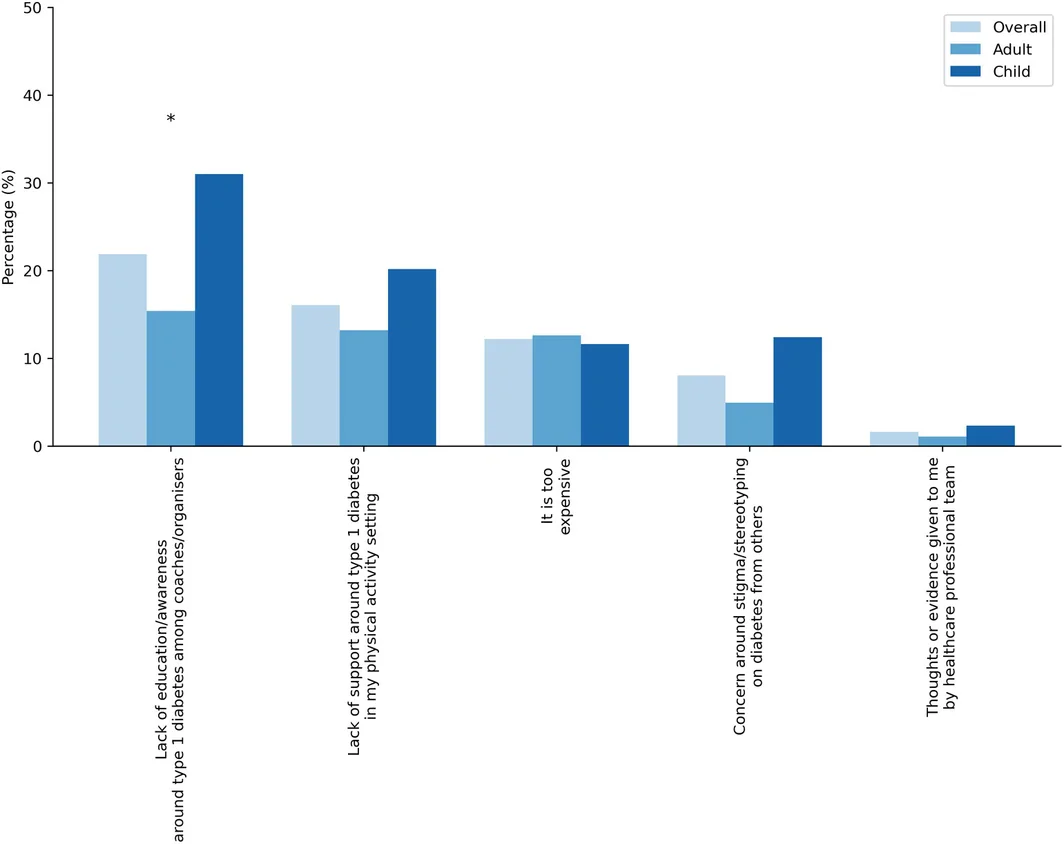
External barriers to physical activity. Significant differences between adults and parent‐proxy responses for children are shown with a * where parent‐proxy responses for children were more likely to report reason compared to adults.

Relating to external factors, approximately one‐fifth of respondents indicated a lack of education or awareness among coaches and organisers (21.9%), and 16.1% cited insufficient support in their physical activity settings. Stigma or stereotyping was reported as a barrier to physical activity by 8.0% and negative messaging from healthcare professionals by only 1.6%. Children, as reported by their parents, were more likely to report a lack of education or awareness among coaches and organisers (31.0% vs. 15.4%, *p* = 0.001, *adjusted p*‐value = 0.006).

As a secondary analysis, we examined whether reported barriers differed according to the use of diabetes technology. Comparisons between participants using any diabetes technology (insulin pump, continuous glucose monitoring or closed‐loop systems) and those not using these technologies revealed no statistically significant differences in reported barriers. Clinical barriers, including fear of hypo‐/hyper‐glycaemia (45.7% vs. 46.9%) and complexity of diabetes management (40.0% vs. 37.0%), were similarly prevalent across groups.

### 3.4 External experiences influencing physical activity

A substantial proportion of participants reported experiencing stigma related to type 1 diabetes and physical activity.

Overall, 73% of participants reported experiencing other people being ignorant about type 1 diabetes at some point. This was reported to happen ‘regularly’ or ‘very often’ by 27% of participants (21% of adults and 36% of children as reported by their parents). Approximately 50% of respondents had heard comments relating to people living with type 1 diabetes not being able to be physically active; this was reported to happen regularly and very often in 7% of participants (5% of adults, 10% children as reported by their parents). Similarly, 58% of participants (51% and 68% for adults and children, respectively) have experienced coaches, physical education teachers or gym instructors expecting less of them because of their type 1 diabetes. This was reported to happen regularly or very often in 6% of respondents, with higher rates in children as reported by their parents compared with adults (2% adults vs. 12% children as reported by their parents). Forty‐seven percent of participants (50% adults, 41% children as reported by their parents) have had people comment on body image in relation to type 1 diabetes. This was reported to happen regularly or very often in 10% of both adults and children as reported by their parents. Comments about diabetes technology during physical activity were reported by 73% of participants, with identical rates observed in both adults and children as reported by their parents; these typically involved unsolicited remarks from others about CGM or pump use or being singled out for having diabetes or a pump. Because this was a closed‐response item, further illustrative examples are presented later in the manuscript with the open‐ended data. Over half of the participants (48% and 59% for adults and children as reported by their parents, respectively) had received comments about type 1 diabetes being their own fault. This was common in 12% of participants (8% of adults and 18% of children as reported by their parents). Further breakdown of results are shown in Figure 4.

**FIGURE 4:**
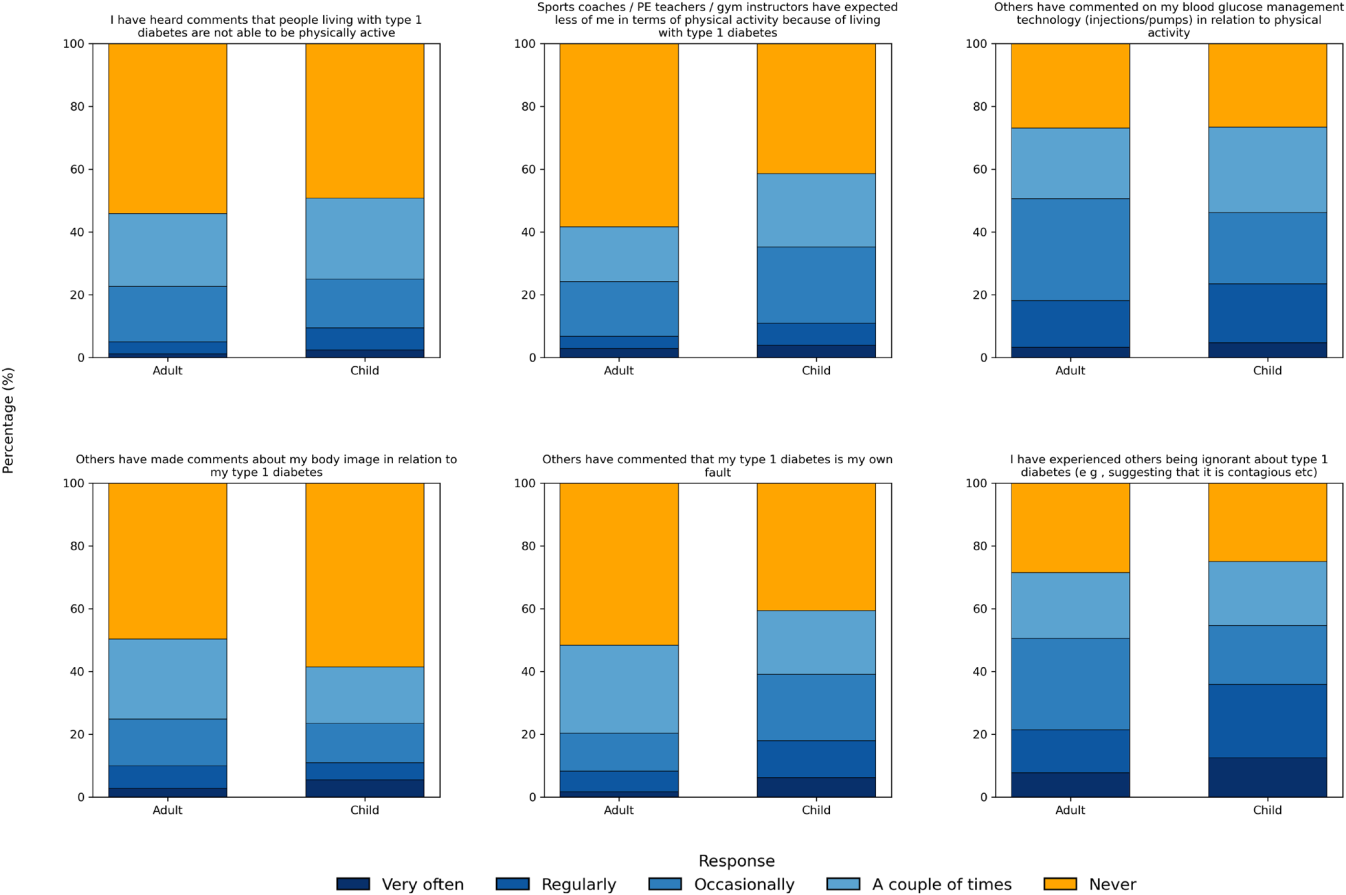
Participants experiences relating to perceived stigma and negative comments about type 1 diabetes. Stacked bar charts show the percentage of respondents who reported different experiences of stigma, stereotyping, or negative comments, stratified by adults with type 1 diabetes and parent‐proxy responses for children with type 1 diabetes. Responses are colour‐coded by reported frequency.

In relation to support from others, 42% of participants (48% of adults and 31% of children as reported by their parents) reported that coaches, physical education teachers or gym instructors had never demonstrated any knowledge of type 1 diabetes in the context of physical activity. Only 37% of participants overall reported receiving regular or frequent support from their healthcare team regarding physical activity. This was notably higher among children as reported by their parents (57%) compared with adults (24%).

Additionally, a significant proportion of respondents reported difficulty accessing relevant information: One‐third of adults (33%) and nearly a quarter of children as reported by their parents (23%) stated they were unable to find accessible guidance on physical activity in relation to type 1 diabetes. A more detailed breakdown of these findings is presented in Figure 5.

**FIGURE 5:**
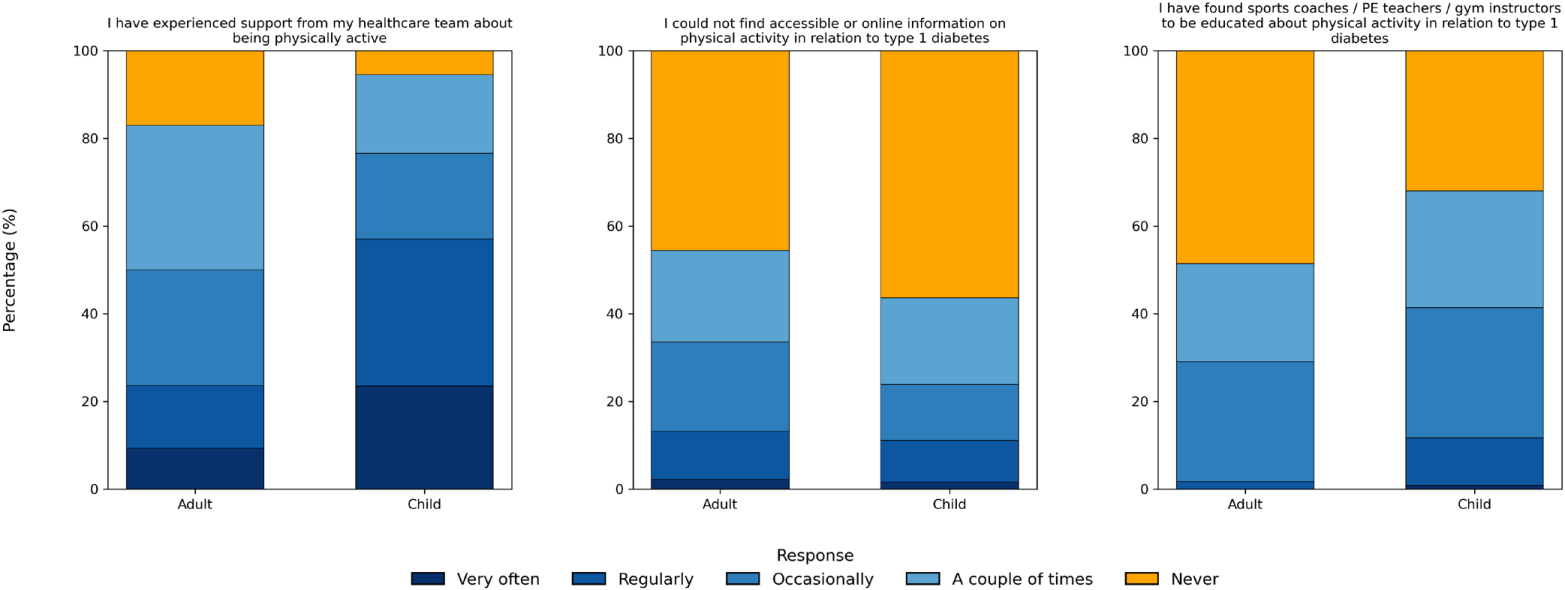
Participants experience of support and information accessibility. Stacked bar charts show the percentage of respondents who reported receiving support or being able to access information about physical activity and type 1 diabetes, stratified by adults with type 1 diabetes and parent‐proxy responses for children with type 1 diabetes. Responses are colour‐coded by reported frequency.

### 3.5 Qualitative insights into factors influencing physical activity

Thematic analysis of free‐text survey responses identified key barriers and motivators influencing physical activity in people with type 1 diabetes.

#### 3.5.1 Barriers to physical activity

Barriers were grouped into three main areas: diabetes‐specific, interpersonal and general. Diabetes‐specific challenges were most frequently cited, with many participants pointing to blood glucose fluctuations and the unpredictability of diabetes as major obstacles. Fear of hypoglycaemia was a common concern (‘*having to stop activities when your blood sugar is too low*’.), alongside frustration with glycaemic instability (‘*it makes me very angry that they are so unreliable!*’). The logistical burden of ongoing diabetes management, especially the need for constant planning, was seen to limit spontaneity (‘it's hard to always have to m plan ahead, can't be spontaneous’). These diabetes‐specific concerns were reported across both groups, although adults more often described the cumulative burden of self management, whereas parent‐proxy responses more frequently reflected challenges related to supervision and safety during activities.

Social barriers often involved a lack of understanding from physical activity providers. Participants described being dismissed or misunderstood by coaches, teachers and gym staff (‘*sometimes people see managing t1 diabetics as too much trouble and not worth the effort*’.). Experiences of stigma and stereotyping were also reported: ‘*comments about me being a recreational drug user because I've had to do injections in front of teammates*’. These social barriers included negative experiences of comments about diabetes technology during physical activity such as ‘Strangers commenting on GCM and pump’ or being singled out for having diabetes and a pump.

Participants also reported common barriers such as lack of time, poor weather, low energy and limited motivation, often exacerbated by the complexities of diabetes management. These more general barriers were more often reported by adults.

#### 3.5.2 Motivators for physical activity

Motivators for physical activity clustered into health and fitness, and psychological or social benefits. Many participants described improved physical and mental health as major drivers. The positive impact of activity on diabetes management was highlighted as a source of motivation (‘*I have found that I can see in my blood glucose management when I am regularly active and when I am not and that gives me motivation to get more active*’.). Others were motivated by body image, weight management and maintaining general fitness. Although reported in both adults and parent‐proxy responses for children, motivation relating to both long‐ and short‐term health outcomes was more common in adult responses.

Psychological and social factors also encouraged participation, including enjoyment (‘above all, I participate in my chosen sports because of enjoyment. It all puts a smile on my face’), social connection (‘*it's sociable and you meet like‐minded people*’) and personal challenge (‘I enjoy a challenge and seeing reward for my training’.). These aspects were reported across both groups; however, enjoyment was more commonly reported in parent‐proxy responses.

In summary, these findings illustrate the diverse and interconnected personal, social and logistical factors shaping physical activity for people with type 1 diabetes, with barriers and motivators often overlapping and context‐specific.

### 3.6 Synthesis of findings

Figure 6 presents a synthesis of key barriers and enablers to physical activity in people with type 1 diabetes, integrating findings from both the COM‐B model and the socioecological model (SEM). This synthesis maps behavioural influences across domains of capability, opportunity and motivation, and across structural levels from the individual to the policy level. While some barriers relate to individual factors such as knowledge and confidence in managing blood glucose during exercise, many respondents described challenges that reflect broader systemic issues. These include limited awareness among coaches and teachers, experiences of stigma and practical barriers such as cost and access, highlighting the need for multilevel, system‐informed approaches.

**FIGURE 6:**
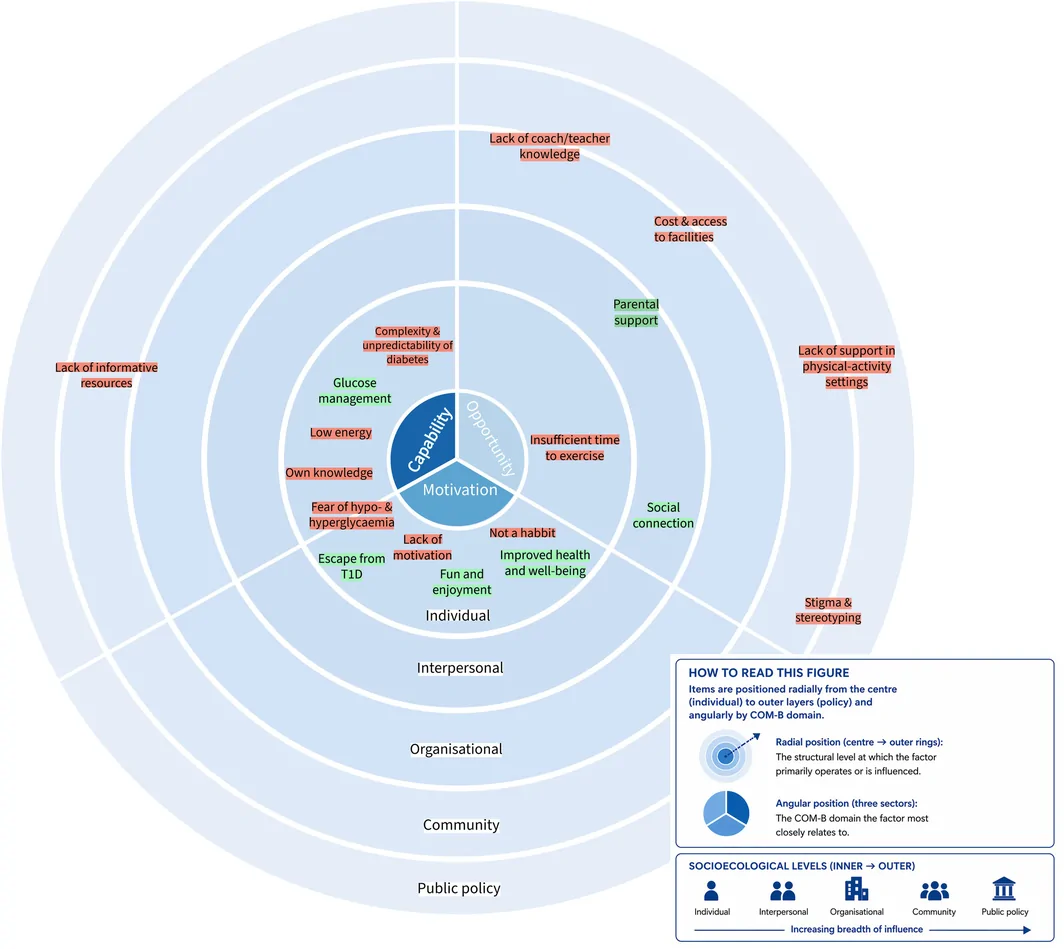
Summary of barriers and enablers to physical activity in individuals with type 1 diabetes. Barriers and enablers to physical activity in people with type 1 diabetes mapped to the COM‐B model and socioecological framework. This figure presents the synthesised findings from a survey of adults with type 1 diabetes and parents of children with type 1 diabetes, displaying key barriers (red), enablers (green), influencing physical activity. Factors are positioned according to the COM‐B behavioural model domains of capability, opportunity and motivation, and are further layered across the socioecological model levels: Individual, interpersonal, organisational, community and public policy. The positioning of each label reflects the dominant behavioural determinant and the structural level at which the factor operates.

Drawing on the behaviour change wheel and theoretical domains framework, several intervention strategies are suggested. These include: education and training to support both people with type 1 diabetes and physical activity providers; enablement strategies such as personalised planning tools, peer mentoring, and parent or carer support networks; environmental restructuring to improve access and inclusivity in physical activity settings; and persuasive and modelling approaches to challenge stigma and promote more positive diabetes‐related identities.

## 4 DISCUSSION

This survey provides one of the first UK‐based comparisons of barriers and enablers to physical activity among adults and parent‐proxy responses for children living with type 1 diabetes. By explicitly recruiting adults and using parent‐proxy responses for children, our study provides a uniquely comprehensive and comparative view. While individual‐level barriers to physical activity such as fear of hypoglycaemia and low motivation remain significant, particularly in adults, our findings highlight the powerful and under‐recognised impact of external factors such as stigma, negative expectations and lack of support from coaches and teachers. This supports a shift in focus from purely individual self management to addressing wider social and structural challenges.

Our data show individual‐level barriers such as fear of hypoglycaemia and management complexity persist despite advances in diabetes technology. This is emphasised in our secondary analyses comparing users and non‐users of diabetes technologies, including closed‐loop systems, which demonstrated no significant differences in reported barriers to physical activity. Clinical concerns such as fear of hypoglycaemia and the complexity of diabetes management remained prevalent across groups, suggesting that technological advancements alone may not be sufficient to address these core barriers.

Among children and adolescents (by parent‐proxy), the dominant barriers are external. Particularly limited support and understanding from coaches, physical education staff and school environments. This echoes previous work of Dash and colleagues^9^ and extends it by demonstrating the scale of these challenges in a large UK sample, highlighting the central role of social and institutional contexts in shaping physical activity participation.

Previous work suggests that parent‐proxy and child self report perspectives on physical activity in type 1 diabetes are related but not interchangeable, with parents more likely to emphasise supervision and safety, and children focusing on enjoyment and social participation.^23^, ^24^ Consistent with this, our findings showed that while diabetes‐specific barriers such as fear of hypoglycaemia were reported across both groups, adult responses more often reflected the cumulative burden of self management, whereas parent‐proxy responses emphasised supervision, safety and enjoyment. Evidence from Parent and colleagues^25^ further supports this interpretation, demonstrating that fear of hypoglycaemia is shared between parents and children but does not necessarily correspond to lower activity levels, suggesting that reported barriers may reflect vigilance rather than inactivity.

Our findings build on recent qualitative work exploring physical activity experiences across the lifespan in type 1 diabetes,^20^ which highlights the pervasive role of stigma, institutional misunderstanding and exclusion in physical activity settings. In this context, a key contribution of the present study is the demonstration that these experiences are not only present but highly prevalent at a national level, and comparable across adults and children (via parent‐proxy responses). A novel and concerning finding of this study is the prominence of stigma and stereotyping as significant barriers to physical activity among individuals with type 1 diabetes. While stigma, including blame, negative judgement, exclusion and discrimination, has been described in broader type 1 diabetes contexts,^26^ previous reports have not quantified stigma specifically in physical activity and sport settings. Our results demonstrate its direct and detrimental impact on physical activity engagement. Stigma has the potential to undermine social relationships, self‐identity, emotional wellbeing and diabetes self management. It may also lead individuals to conceal their condition. This is supported by systematic review evidence showing that stigma adversely affects self care practices, uptake of diabetes technologies and willingness to disclose one's condition.^17^

Another novel finding is the large and previously under‐reported gap in regular healthcare provider support for physical activity between age groups (24% of adults vs. 57% of children as reported by their parents). This suggests that although paediatric diabetes teams have integrated physical activity support effectively, adult services lag substantially, potentially reinforcing drop‐off in physical activity engagement after transition from paediatric to adult care. Interventions to embed regular physical activity counselling for adults with type 1 diabetes at routine reviews are urgently needed, alongside tools and incentives enabling busy providers to prioritise physical activity support.

In addition to stigma, lack of support and knowledge among coaches, physical education teachers and gym instructors remains a key barrier. This is an issue highlighted in both our current and previous research.^27^ This challenge appears particularly pronounced among children and adolescents, who rely on adults for decision making and safety during physical activity. Sadly, some participants were resigned to the lack of awareness among providers, perceiving it as an accepted norm. These experiences and expectations from coaches and other physical activity providers reinforce the need for targeted community‐level interventions to enhance diabetes literacy among physical activity providers. Sustained action and co‐designed training with people with type 1 diabetes, families and key sports/education stakeholders will be critical to deliver lasting, equitable benefits. Such initiatives are essential not only to improve practical competence but also to foster more inclusive, supportive and equitable environments for individuals with type 1 diabetes across the lifespan. Together, these findings extend existing qualitative and international evidence by providing UK‐specific, comparative data that highlight both shared and age‐specific barriers to physical activity in type 1 diabetes.

### 4.1 Study limitations

This study has several limitations. The use of a convenience sample recruited via a diabetes charity network may have introduced selection bias, with potential overrepresentation of individuals more engaged with diabetes organisations and a high proportion of technology users^20^ limiting generalisability. However, the ethnic distribution of the sample was broadly in line with UK population averages, suggesting some degree of representativeness in this respect. The gender distribution should also be considered, with a higher proportion of female respondents among adults and a greater representation of boys in parent‐proxy responses for children, which may introduce bias if experiences of physical activity, stigma or support differ by gender.

All data were self reported and may be influenced by recall or social desirability bias. Parent‐proxy responses for children may not fully reflect children's own experiences, and no data were collected on the responding parent, limiting the ability to explore the influence of parent characteristics on responses.

The survey did not include a measure that allowed accurate quantification of overall physical activity levels or classification against recommended thresholds. Although frequency of participation across a range of activity types was captured, total physical activity volume and guideline adherence could not be assessed. As a result, we were unable to determine how representative the sample was in relation to overall physical activity participation or examine how reported barriers and enablers related to actual behaviour.

Despite these limitations, the large sample and mixed‐methods design provide valuable insights into the multilevel factors influencing physical activity in people with type 1 diabetes and inform priorities for future research and intervention development.

### 4.2 Research and policy implications

These findings highlight the need for multilevel, tailored interventions to support physical activity in people with type 1 diabetes. For adults, strategies should focus on building confidence, addressing hypoglycaemia fears and supporting self management through education and personalised tools. Importantly, the marked reduction in healthcare professional support for physical activity in adults compared with children highlights a critical gap in service provision. While paediatric care appears to embed physical activity support more routinely, adults are often left without structured guidance, indicating a need to strengthen healthcare professional knowledge, confidence and delivery of physical activity support within adult diabetes services. For children and adolescents, the emphasis should be on creating supportive environments, with targeted training for coaches and teachers to reduce stigma and improve understanding.

Interventions should operate across individual, interpersonal, organisational and community levels, supported by policy measures such as embedding diabetes education in schools and sports settings. At a systems level, there is a need to better integrate physical activity support into routine diabetes care pathways, including training, resources and incentives for healthcare professionals. Future research should prioritise co‐designed approaches with people with type 1 diabetes, families and key stakeholders to ensure interventions are relevant, acceptable and sustainable.

## 5 CONCLUSION

Collectively, these findings underscore the urgent need for multilevel, evidence‐informed interventions to address stigma and stereotyping in type 1 diabetes. Educational initiatives targeting the general public, healthcare professionals, educators and sports coaches are essential to challenge misconceptions and promote understanding of type 1 diabetes. Regular physical activity counselling should be embedded as a standard component of diabetes care for all age groups, closing current gaps in support that disproportionately affect adults. Training for coaches and school staff must be mandated and co‐designed with people living with type 1 diabetes to ensure environments are supportive and inclusive. Policy measures to enforce antidiscrimination legislation and ensure reasonable adjustments in all settings are also critical. Addressing stigma is not only a matter of individual wellbeing but also a fundamental issue of equity and inclusion for people living with type 1 diabetes.

## FUNDING INFORMATION

This research received no external funding.

## 6 CONFLICT OF INTEREST STATEMENT

All the authors declare that they have no competing interest, financial or otherwise. The views expressed are those of the authors and not necessarily those of the NHS, the NIHR or the Department of Health and Social Care.

## ETHICS STATEMENT

This study involved secondary analysis of an existing dataset. The study was conducted according to the ethical guidelines of the declaration of Helsinki.

## Supporting information


**Data S1:** JDRF physical activity and type 1.

## Data Availability

This study involved secondary analysis of an existing dataset. Data available from third party upon reasonable request.
