# Supplementary material for: Barriers and enablers to physical activity in type 1 diabetes: A mixed‐methods study of adults and parent‐reported child perspectives in the UK
Source: Diabet Med. 2026 Jun 19;43(10):e70378. doi: 10.1111/dme.70378 (PMC13643639; doi:10.1111/dme.70378)
Supplement: Supplementary file 1 [file DME-43-e70378-s001.docx]

# JDRF Physical Activity and Type 1

**Page exit logic:** Disqualify if no experience of Diabetes 1**IF:** #1 Question "**Do you have any of the following health conditions?**" is one of the following answers ("Type 2 diabetes","None of the above") **THEN:** Disqualify and display: "Thank you for your availability and for taking the time to consider participating in our survey. Unfortunately, after reviewing your information, it appears that you do not qualify for this particular survey. We apologize for any inconvenience this may have caused and appreciate your understanding. Again, thank you for your time and consideration."

**Thank you for taking the time to participate in this survey. It is being carried out by nfpResearch on behalf of a diabetes charity.**

**In the following questions, we will be asking you about movement with type 1 diabetes. This can include any activity you deem to be physical, such as walking the dog, doing household chores and structured exercise such as sports, amongst other things.

The survey will take around 10 minutes to complete. We are interested in your honest and instinctive responses and there are no right or wrong answers. Your responses will be fully anonymised and no personally identifiable data will be collected. Thank you for your time. 

If you want to fill in this survey on behalf of a child with type 1 diabetes in your care, please fill in this survey [nfpResearch to add link once this survey has been signed off].**

#### 1) ****Do you have any of the following health conditions?*****

Type 1 diabetes

Type 2 diabetes

None of the above

### 2) ****What is your age?*****

If you want to fill in this survey on behalf of a child with type 1 diabetes in your care, please fill in this survey [nfpResearch to add link]

**Page exit logic:** Skip / Disqualify Logic**IF:** #2 Question "**What is your age?**" is less than "18" **THEN:** Disqualify and display: "Thank you for your availability and for taking the time to consider participating in our survey. Unfortunately, after reviewing your information, it appears that you do not qualify for this particular survey because you need to be at least 18 to take part in it. Feel free to share the survey link to the adult responsible of your care, so that he can fill the survey on your behalf. We apologize for any inconvenience this may have caused and appreciate your understanding. Again, thank you for your time and consideration."

### 3) ****Please let us know in the textbox below what physical activities you engage in.****

#### 4) ****How often do you take part in the following activities?****

|  | **Daily** | **Several times a week** | **Several times a month** | **Less than once a month** | **A couple of times per year** | **Never** |
| --- | --- | --- | --- | --- | --- | --- |
| Walking |  |  |  |  |  |  |
| Gardening |  |  |  |  |  |  |
| Housework |  |  |  |  |  |  |
| Shopping |  |  |  |  |  |  |
| DIY |  |  |  |  |  |  |
| Gym or exercise classes |  |  |  |  |  |  |
| Sports sessions/games |  |  |  |  |  |  |
| Jog / run |  |  |  |  |  |  |
| Cycling |  |  |  |  |  |  |
| At home exercises |  |  |  |  |  |  |
| Yoga / Pilates |  |  |  |  |  |  |
| Hiking |  |  |  |  |  |  |
| Dancing |  |  |  |  |  |  |

### 5) ****Please tell us in your own words below what motivates you to engage in physical activity.****

### 6) ****Please tell us in your own words below what, if anything, discourages you from engaging in physical activity.****

#### 7) ****Please indicate whether you agree or disagree with the following statements.****

|  | **Strongly agree** | **Agree** | **Neither agree nor disagree** | **Disagree** | **Strongly disagree** | **Don't know / not sure** |
| --- | --- | --- | --- | --- | --- | --- |
| I would like to engage in more physical activity |  |  |  |  |  |  |
| I don’t feel like I need to engage in more physical activity |  |  |  |  |  |  |
| It is important for people living with type 1 diabetes to engage in physical activity |  |  |  |  |  |  |
| I think that people living with type 1 diabetes cannot be physically active |  |  |  |  |  |  |
| Physical activity can be helpful with blood glucose management |  |  |  |  |  |  |
| I feel like I don’t know enough about physical activity and type 1 diabetes |  |  |  |  |  |  |
| I would like to get informed about how I could engage in physical activity |  |  |  |  |  |  |
| I don’t feel I have the appropriate medical technology to help me be physically active |  |  |  |  |  |  |
| I feel that the knowledge of type 1 diabetes in those organising exercise is not sufficient enough to support me |  |  |  |  |  |  |

#### 8) ****What are reasons for you to engage in physical activity?****

Please select up to 5 reasons and describe other reasons below if needed.

To manage my glucose level

I have fun / enjoy it

To escape from type 1 diabetes

It makes me feel good

For my general health

I should be active / out of guilt

It is good for my mental health

Financial goals / career

For social contact (friendships, etc.)

For my body image

To commute to work

For the necessities of my daily life (housework, shopping, etc.)

Sense of accomplishment/ physical challenge

Other (please specify)

None of the above

### 9) Other reason (please specify):

#### 10) ****What, if anything, keeps you from engaging in more physical activity?****

Please select up to 5 reasons and describe other reasons below if needed.

Complexity of type 1 diabetes management

Lack of education/awareness around type 1 diabetes among coaches/organisers

Lack of support around type 1 diabetes in my physical activity setting

Fear of hypo-/hyperglycaemia

Concern around stigma/stereotyping on diabetes from others

Lack of motivation

I don't enjoy it

I have no time to engage in (more) physical activity

I have never built a habit of engaging in physical activity

Thoughts or evidence given to me by healthcare professional team

It is too expensive

Other (please specify)

None of the above

### 11) Other reason (please specify):

#### 12) ****Have you ever experienced any of the following?****

For each statement, please select how often you have experienced it.

|  | **Never** | **A couple of times** | **Occasionally** | **Regularly** | **Very often** |
| --- | --- | --- | --- | --- | --- |
| I have heard comments that people living with type 1 diabetes are not able to be physically active |  |  |  |  |  |
| Sports coaches / PE teachers / gym instructors have expected less of me in terms of physical activity because of living with type 1 diabetes |  |  |  |  |  |
| Others have commented on my blood glucose management technology (injections/pumps) in relation to physical activity |  |  |  |  |  |
| Others have made comments about my body image in relation to my type 1 diabetes |  |  |  |  |  |
| I have heard comments expecting me to be inactive or lazy because of living with type 1 diabetes |  |  |  |  |  |
| Others have commented that my type 1 diabetes is my own fault |  |  |  |  |  |
| I have experienced others being ignorant about type 1 diabetes (e.g., suggesting that it is contagious etc) |  |  |  |  |  |
| I have experienced support from my healthcare team about being physically active |  |  |  |  |  |
| I could not find accessible or online information on physical activity in relation to type 1 diabetes |  |  |  |  |  |
| I have found sports coaches / PE teachers / gym instructors to be educated about physical activity in relation to type 1 diabetes |  |  |  |  |  |

#### 13) ****Please let us know if you have experienced any of the following by any of the groups below.**** ****I have experienced****

|  | **Family** | **Friends** | **Colleagues / Acquaintances** | **Healthcare professionals** | **Sports instructors / PE teachers** |
| --- | --- | --- | --- | --- | --- |
| comments about your body image in relation to your type 1 diabetes |  |  |  |  |  |
| comments that your type 1 diabetes is your own fault |  |  |  |  |  |
| support to be physically active |  |  |  |  |  |
| comments on your blood glucose management technology (injections/pumps) in relation to physical activity |  |  |  |  |  |
| comments that people living with type 1 diabetes are not able to be physically active |  |  |  |  |  |
| support about being physically active |  |  |  |  |  |

### 14) ****Have you experienced anything else that has had an impact on your attitudes towards physical activity?**** ****Feel free to describe your experience in the box below.****

#### 15) ****Do you think any of the following experiences have impacted your attitudes towards physical activity****

|  | **Very much** | **A little** | **Not really** | **Not at all** | **Don't know / Not sure** |
| --- | --- | --- | --- | --- | --- |
| Sports coaches / PE teachers / gym instructors have expected less of me in terms of physical activity because of living with type 1 diabetes |  |  |  |  |  |
| Others have commented that my type 1 diabetes is my own fault |  |  |  |  |  |
| I could not find accessible or online information on physical activity in relation to type 1 diabetes |  |  |  |  |  |
| I have experienced support from my healthcare team about being physically active |  |  |  |  |  |
| Others have made comments about my body image in relation to my type 1 diabetes |  |  |  |  |  |
| I have found sports coaches / PE teachers / gym instructors to be educated about physical activity in relation to type 1 diabetes |  |  |  |  |  |
| I have heard comments expecting me to be inactive or lazy because of living with type 1 diabetes |  |  |  |  |  |
| I have heard comments that people living with type 1 diabetes are not able to be physically active |  |  |  |  |  |
| Others have commented on my blood glucose management technology (injections/pumps) in relation to physical activity |  |  |  |  |  |
| I have experienced others being ignorant about type 1 diabetes (e.g., suggesting that it is contagious etc) |  |  |  |  |  |

#### 16) ****Please indicate to what extent the following list of people have influenced your attitudes towards physical activity with type 1 diabetes.****

| Friends | 1 ________________________[__]_____________________________ 10 |
| --- | --- |
| Teachers/Lecturers / PE teachers | 1 ________________________[__]_____________________________ 10 |
| Family | 1 ________________________[__]_____________________________ 10 |
| Diabetes specialist psychologist | 1 ________________________[__]_____________________________ 10 |
| Sports coaches / gym instructors | 1 ________________________[__]_____________________________ 10 |
| Type 1 Athletes/Social Media Advocates | 1 ________________________[__]_____________________________ 10 |
| Diabetes dieticians | 1 ________________________[__]_____________________________ 10 |
| Diabetes nurses | 1 ________________________[__]_____________________________ 10 |
| Peers/team mates / Colleagues | 1 ________________________[__]_____________________________ 10 |
| GP practice specialist nurses | 1 ________________________[__]_____________________________ 10 |
| Diabetes Consultants | 1 ________________________[__]_____________________________ 10 |
| GPs | 1 ________________________[__]_____________________________ 10 |

#### 17) ****Have you experienced any situation where you felt others had stereotypes about type 1 diabetes and physical activities?*****

Yes

No

Not sure

**Page entry logic:** This page will show when: #17 Question "**Have you experienced any situation where you felt others had stereotypes about type 1 diabetes and physical activities?**" is one of the following answers ("Yes")

#### 18) ****Do you feel these situations had an impact on:****

| Your social and family life | 1 ________________________[__]_____________________________ 10 |
| --- | --- |
| Your general health and well-being | 1 ________________________[__]_____________________________ 10 |
| You engaging in physical activity | 1 ________________________[__]_____________________________ 10 |
| Your mental health | 1 ________________________[__]_____________________________ 10 |

#### 19) ****Where are you most likely to seek information on engaging in physical activity?****

Please tick all that apply.

Doctor or another clinician

Diabetes specialist nurse

Dietician

Through groups for people living with / affected by type 1 diabetes

Internet searches / online forums

Charities/ charity websites

Friends and family

YouTube

Twitter

Instagram

Facebook

TikTok

Type 1 Athletes/ Social Media Advocates

Type 1 specific exercise education programmes (EXTOD/ SEREN Active)

WhatsApp groups

Diabetes specialist psychologist

Other (please specify):

None of the above

#### 20) ****From the following list, what are important things you would like a diabetes charity to be doing.****

 Please tick one box for each statement.

|  | **Very important** | **Important** | **Neither important nor unimportant** | **Quite unimportant** | **Very unimportant** | **Don't know / Not sure** |
| --- | --- | --- | --- | --- | --- | --- |
| Provide information on physical activity options for people living with type 1 diabetes |  |  |  |  |  |  |
| Lobby the NHS for better treatments at the hospital level |  |  |  |  |  |  |
| Lobby the government to fund research into type 1 diabetes and physical activity |  |  |  |  |  |  |
| Lobby the government to improve the prioritisation of diabetes |  |  |  |  |  |  |
| Bring people with diabetes together |  |  |  |  |  |  |
| Be a source of personal advice and support |  |  |  |  |  |  |
| Educate the general public on type 1 diabetes and physical activity |  |  |  |  |  |  |
| Educate sports instructors / gym staff etc. on type 1 diabetes and physical activity |  |  |  |  |  |  |
| Educate healthcare professionals on type 1 diabetes and physical activity |  |  |  |  |  |  |

### ****The following questions are about your experience with diabetes.****

#### 21) ****How long ago were you diagnosed with type 1 diabetes?****

Less than one year ago

1-2 years ago

3-5 years ago

6-10 years ago

11-20 years ago

More than 20 years ago

I don't know / can't remember

### 22) ****How old were you when you were diagnosed with type 1 diabetes?****

#### 23) ****Which of the technologies below do you currently use to manage your condition or have used in the past?****

Please select all that apply.

|  | **Currently** | **In the past** |
| --- | --- | --- |
| Insulin pump |  |  |
| Closed loop technology |  |  |
| Injections |  |  |
| Continuous glucose monitoring (CGM) |  |  |
| Flash glucose monitoring (e.g., Freestyle Libre) |  |  |
| None of the above |  |  |

#### 24) ****How satisfied or dissatisfied do you feel about your current blood glucose management?****

Very satisfied

Somewhat satisfied

Neither satisfied nor dissatisfied

Somewhat dissatisfied

Very dissatisfied

Don’t know / Not sure

#### 25) ****Who do you discuss your type 1 diabetes (or the diabetes of the child you care for) with?****

Please select all that apply.

GP, or practice nurse at GP surgery

Specialist diabetes team, or specialist diabetes doctor, or specialist diabetes nurse

Other (please specify):

No one

Piping: Piped From Question 25. (**Who do you discuss your type 1 diabetes (or the diabetes of the child you care for) with?**)

#### ****How often do you discuss your type 1 diabetes with****

|  | **Once a month or more** | **Every 2-3 months** | **Every 4-6 months** | **Every 7-12 months** | **Every 1-2 years** | **Less than once every 2 years** | **Don’t know / not sure** |
| --- | --- | --- | --- | --- | --- | --- | --- |

Piping: Piped From Question 25. (**Who do you discuss your type 1 diabetes (or the diabetes of the child you care for) with?**)

#### ****Would you like to engage more with the GP or specialist about your type 1 diabetes?****

|  | **Yes definitely** | **Yes probably** | **Probably not** | **Fine as is** | **Don’t know / not sure** | **Never meet the GP or specialist** |
| --- | --- | --- | --- | --- | --- | --- |

#### 26) ****Are your day-to-day activities limited because of a health problem or disability which has lasted or is expected to last, at least 12 months?****

Please include problems related to old age.

Yes, limited a lot by type 1 diabetes

Yes, limited a lot by type 1 diabetes and other condition(s)

Yes limited a lot by conditions other than type 1 diabetes

Yes, limited a little by type 1 diabetes

Yes, limited a little by type 1 diabetes and other condition(s)

Yes limited a little by conditions other than diabetes

No

#### 27) ****Do you have any of the following health conditions?****

Please select all that apply.

Cystic fibrosis

Chronic obstructive pulmonary disease (COPD)

High blood pressure

Migraines

Epilepsy

Addison disease

Celiac disease - sprue (gluten-sensitive enteropathy)

Dermatomyositis

Graves disease

Hashimoto thyroiditis

Multiple sclerosis

Myasthenia gravis

Pernicious anemia

Reactive arthritis

Rheumatoid arthritis

Sjögren syndrome

Systemic lupus erythematosus

Other (please specify):

None of the above

### ****You’ve nearly completed the survey. Just a couple of questions about you.****

#### 28) ****How do you describe your gender?****

Man/male

Woman/female

Non-binary

Other (please specify):

Prefer not to say

#### 29) ****Where do you live?****

Channel Islands

East of England

East Midlands

London

North East

North West

Northern Ireland

Scotland

South East

South West

Wales

West Midlands

Yorkshire/Humberside

None of the above

#### 30) ****Please indicate to which occupational group the Chief Income Earner in your household belongs, or which group fits best.****

This could be you: the Chief Income Earner is the person in your household with the largest income.
If the Chief Income Earner is retired and has an occupational pension please answer for their most recent occupation. If the Chief Income Earner is not in paid employment but has been out of work for less than 6 months, please answer for their most recent occupation.

Semi or unskilled manual work (e.g. Manual workers, all apprentices to be skilled trades, Caretaker, Park keeper, non-HGV driver, shop assistant)

Skilled manual worker (e.g. Skilled Bricklayer, Carpenter, Plumber, Painter, Bus/ Ambulance Driver, HGV driver, AA patrolman, pub/bar worker, etc)

Supervisory or clerical/ junior managerial/ professional/ administrative (e.g. Office worker, Student Doctor, Foreman with 25 employees, salesperson, etc).

Intermediate managerial/ professional/ administrative (e.g. Newly qualified (under 3 years) doctor, Solicitor, Board director small organisation, middle manager in large organisation, principle officer in civil service/local government)

Higher managerial/ professional/ administrative (e.g. Established doctor, Solicitor, Board Director in a large organisation (200 employees, top level civil servant/public service employee)

Student

Casual worker not in permanent employment

Homemaker

Retired and living on state pension

Unemployed, or not working due to long-term sickness

Full-time carer of other household member

#### 31) ****What is your ethnic group? Choose one option that best describes your ethnic group or background****

Indian

Pakistani

Bangladeshi

Chinese

Any Other Asian background (Please specify): *

Caribbean

African

Any Other Black/African/Caribbean background (Please specify): *

White and Black Caribbean

White and Black African

White and Asian

Any Other Mixed/Multiple ethnic background (Please specify): *

English, Welsh, Scottish, Northern Irish or British

Irish

Gypsy, Traveller or Irish Traveller

Any Other White background (Please specify): *

Arab

Any Other ethnic group (Please specify): *

Prefer not to say

#### 32) ****Which of the following options best describes how you think of yourself?****

Asexual

Bisexual

Gay or Lesbian

Heterosexual or straight

Any other sexual identity:

Prefer not to say

#### 33) ****What is your religion?****

Buddhist

Christian (including Church of England, Catholic, Protestant and all other Christian denominations)

Hindu

Jewish

Muslim

Sikh

No religion

Any other religion (please specify): *

Prefer not to say

## Thank You!

### Thank you for taking part in this survey. Your response is very valuable to us.
